# Rhodopsin driven microbial CO_2_ fixation using synthetic biology design

**DOI:** 10.1111/1462-2920.16243

**Published:** 2022-10-20

**Authors:** Weiming Tu, Wei E. Huang

**Affiliations:** ^1^ Department of Engineering Science University of Oxford Oxford UK

Solar energy is the ultimate source of sustainable energy. One‐hour of the sunlight energy received by the earth has more energy than the whole human needs in a year. Microorganisms and plants use sunlight as energy sources to fix CO_2_ and drive biosynthesis of biomass, which is the foundation of the biosphere on the earth. Sunlight energy harvesting is the first crucial step to use the solar energy. Chlorophyll‐based and rhodopsin‐based light harvesting systems are two distinct and absorbance complementary mechanisms (Bryant & Frigaard, 2006; Finkel et al., 2013). Classic chlorophyll‐based systems in photosynthesis have been well documented. The discovery of proteorhodopsin (PR) has changed the view of its role in natural ecosystem (Beja et al., 2000; Beja et al., 2001; Rozenberg et al., 2021). PR is globally abundant and widely distributed in microbes (Beja et al., 2000; Beja et al., 2001; Campbell et al., 2008; Finkel et al., 2013; Fuhrman et al., 2008; Jing et al., 2018; Rusch et al., 2007; Sabehi et al., 2005). A recent survey in the Mediterranean Sea and the Eastern Atlantic Ocean suggested that microbial rhodopsins‐based systems could contribute to the same amount of light energy harvesting as chlorophyll‐based systems (Gomez‐Consarnau et al., 2019). In comparison to chlorophyll‐based photosynthesis that assembles a large and relative complex network of light‐harvesting, a rhodopsin‐based functional activity only involves one single gene encoded membrane protein with retinal.

Rhodopsins are light‐activated ion transporters, including light‐driven pumps and light‐gated channels (Ernst et al., 2014; Rozenberg et al., 2021). Among them, proton‐pumping PR has been well‐studied and documented. It can act as a light activated proton pump, harvesting solar energy to pump out protons for the generation of a proton motive force which supports ATP synthesis (Steindler et al., 2011), biomass growth (Gómez‐Consarnau et al., 2007), substrate uptake (Gómez‐Consarnau et al., 2016), and bacterial survival (Gómez‐Consarnau et al., 2010). The simplicity of rhodopsin offers a new approach to genetically engineering non‐phototrophic cells for harvesting light energy. Engineered *Escherichia coli* with rhodopsin was reported to have a long‐term viability (Song et al., 2019), enhanced biomass growth (Kim et al., 2017) and accelerated target compound biosynthesis (Toya et al., 2022).

The conventional concept of rhodopsin phototrophy is based on light‐driven extra energy supply, such as increasing ATP to promote microbial carbon fixation. It has been reported that PR powered anaplerotic CO_2_ fixation could occur in *Dokdonia* sp. MED134 (Palovaara et al., 2014), and in marine bacteria (Alonso‐Sáez et al., 2010; Beier et al., 2015; Jing et al., 2022; Kirchman et al., 2007; Koedooder et al., 2018; Moran & Miller, 2007; Pinhassi et al., 2016; Smith et al., 2010). Despite microbial rhodopsins representing the most widespread phototrophic system at the genetic level (Finkel et al., 2013), the relationship between rhodopsin and microbial autotrophic carbon fixation is still unclear. It is possible that the significance of rhodopsin in global carbon cycling could be underestimated.

Both ATP and reducing equivalents (e.g., NADH or NADPH) are required for cell growth. We reason that rhodopsin cannot drive the microbial autotrophic CO_2_ fixation pathway on its own because the system lacks electron donor. When an organic compound such as formate was used as the electron donor, *Gloeobacter* rhodopsin‐expressing *Ralstonia eutropha* can increase 20% biomass growth in the light (Davison et al., 2022). Formate is split into CO_2_ and NADH when it gets into *Ralstonia eutropha*. Extra ATP can be produced by the light activated proton pump *Gloeobacter* rhodopsin. Chlorophyll‐based photoautotrophic systems obtain electron donors from light‐driven water splitting, which can be used to generate NADPH. Rhodopsin phototrophy, unlike chlorophyll‐based photosynthesis, is independent of electron transfer and is not involved into any known redox processes (Hassanzadeh et al., 2021). We hypothesised that a closed redox loop can be constructed by integrating rhodopsin with an electron donor. In such a rhodopsin‐based autotrophic system, an electron donor could be an electrode powered by a solar panel, and an electron shuttling molecule such as riboflavin could transfer the electron from the electrode to rhodopsin‐expressing microbes (Davison et al., 2022). In this designed system, a rhodopsin‐based photoelectrosynthetic system could drive autotrophic growth of bacteria using CO_2_ as the sole carbon source, and with light as the only energy input (Figure 1). The proton motive force generated from rhodopsin has been proven to enable microbes to overcome thermodynamically unfavourable processes to reduce NAD^+^ and generate NADH (Tefft & TerAvest, 2019). Then the light‐activated rhodopsins can drive the reversing function of NADH dehydrogenase for the synthesis of NADH from quinones. Rhodopsin is not only a “booster” but also “drive” electron transfer. We therefore established a photoelectrochemical CO_2_ fixation system with engineered *Ralstonia eutropha*, in which the heterogeneously expressing *Gloeobacter* rhodopsin powered both ATP and NADH formation to drive CO_2_ fixation. The engineered *Ralstonia eutropha* accepted electrons from an electrode mediated by an electron shuttle (Davison et al., 2022) (Figure 1). Such Ying (electrons) and Yang (protons) interactions not only power ATP synthesis but also form redox reactions to complete the cycle of biosynthesis.

**FIGURE 1:**
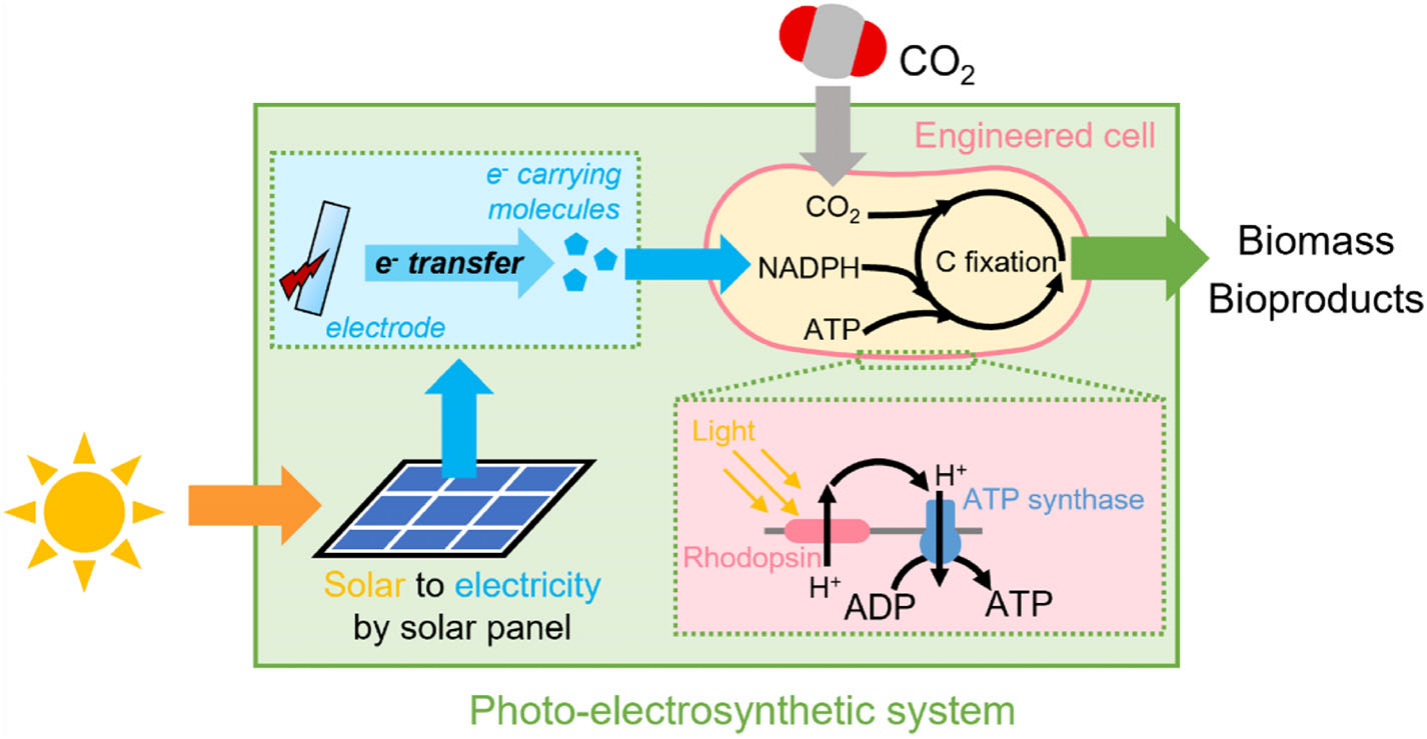
Graphic shows the concept of rhodopsin‐based photoelectrosynthesis, in which light‐activated rhodopsin generates ATP and supports microbes to reverse NADH dehydrogenase to synthesize NADH which is then further converted into NADPH by transhydrogenase.

The rhodopsin‐based CO_2_ fixation system enabled *Ralstonia eutropha* to perform autotrophic growth using light as the only energy source and CO_2_ as the sole carbon source. Since microbial rhodopsin is simple and able to be expressed in many different microbes, such rhodopsin‐based photoelectrosynthesis can be extended to a broad range of microbes for CO_2_ fixation, which could open a new frontier in “rhodopsin‐based photosynthesis.” The light intensity on the solar panel was only 2 μmol m^−2^ s^−2^ to generate ~1.6–1.8 V to drive autotrophic growth of *R. eutropha*‐GR in the photo‐electrosynthetic system (Figure 1). Given that the light toelectricity efficiency in solar panel is usually 20%, the overall solar energy to biomass in photo‐electrosynthetic system was about 4%, which is comparable to conventional chlorophyll‐based photosynthesis (Davison et al., 2022). The design of rhodopsin‐based photosynthesis can be further improved by learning the lessons from chlorophyll‐based system (Ort et al., 2015), including light capture, carbon capture and smart consortium.

Rhodopsins absorb wavelength around 530 nm which is complementary to the chlorophyll system. Directed evolution is able to extend the rhodopsin's light absorption. *Gloeobacter violaceus* rhodopsin was reported to be evolved into 70 variants with absorption maxima shifted by up to ±80 nm (Engqvist et al., 2015). With novel retinal analogues, rhodopsin was able to expand the absorbance band, tailing out to near‐infrared wavelengths (Ganapathy et al., 2017). The quantum efficiency of rhodopsins can reach almost 70% (Govindjee et al., 1990; Yang et al., 2022). Many microbes can take up both CO_2_ and bicarbonate using their various channels, the extra energy supplied by light‐activated rhodopsins may increase the flux of inorganic carbon into cells, enhancing carbon capture efficiency.

Another potential application of rhodopsin is to create a consortium or coculture containing both chlorophyll‐ and rhodopsin‐based microbes. At least 10% of energy from photosynthetically active radiation is lost in chlorophyll‐based photosynthesis due to its weak absorbance in the green band (Zhu et al., 2008). The loss of light utilisation is a key factor for the low efficiency of photosynthesis for CO_2_ reduction. Notably, chlorophyll's absorption spectrum is complemented by rhodopsin's (Walter et al., 2010). Therefore, the consortium or coculture is expected to improve the utilisation of solar energy (Figure 2). The same goal can be accomplished by cloning rhodopsin into chlorophyll‐containing bacteria using synthetic biology design. To address the problem that two photosystems compete for the same photon sources, rhodopsin has been reported to replace photosystem I (PSI) in *Synechocystis* sp. PCC6803, increasing the growth rate by 16% (Chen et al., 2019). The expanded light absorbance of rhodopsin enables the design of synthetic mixed consortium to achieve maximal light capture and CO_2_ fixation per unit of surface area (Figure 2).

**FIGURE 2:**
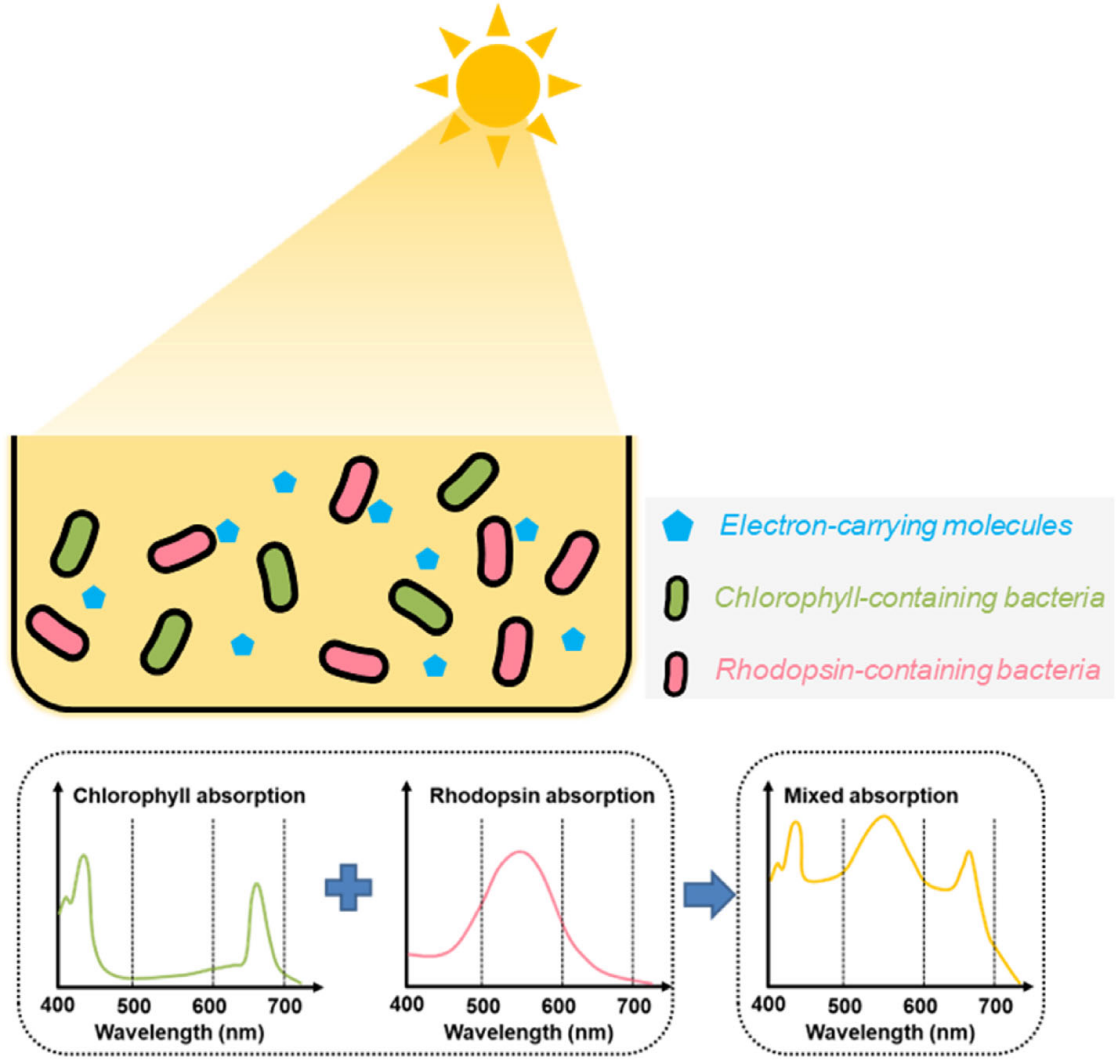
Graphic shows coculture of chlorophyll‐containing microbes and rhodopsin‐containing microbes to maximize solar energy harvesting

Microbial rhodopsins are ubiquitous and multifunctional in the biosphere (Rozenberg et al., 2021). The roles of microbial rhodopsins in nature will be better understood through further studies on molecular microbial ecology and biochemistry (Chazan et al., 2022; Pushkarev et al., 2018). The exploration of rhodopsins is consistently updating our perspectives on their structures, functions and molecular mechanisms. The applications of rhodopsins can lead to new research of photosynthesis via advanced design of synthetic biology (Davison et al., 2022; Inoue et al., 2021), promoting for faster and more efficient biosynthesis and CO_2_ fixation.

## AUTHOR CONTRIBUTIONS

WEH proposed the idea. WEH and WT write the manuscript.

## CONFLICT OF INTEREST

The authors declare no competing financial interest.
